# Cesarean section rate in Iran, multidimensional approaches for behavioral change of providers: a qualitative study

**DOI:** 10.1186/1472-6963-11-159

**Published:** 2011-07-05

**Authors:** Bahareh Yazdizadeh, Saharnaz Nedjat, Kazem Mohammad, Arash Rashidian, Nasrin Changizi, Reza Majdzadeh

**Affiliations:** 1 Department of Epidemiology and Biostatistics, School of Public Health, Tehran University of Medical Sciences, Tehran, Iran; 2 Knowledge Utilization Research Center, Tehran University of Medical Sciences, Tehran, Iran; 3 Department of Health Management and Economics, School of Public Health, Tehran University of Medical Sciences, Tehran, Iran; 4 National Institute of Health Research (NIHR), Tehran, Iran; 5 General Directory of Primary Care Health Center, Ministry of Health and Medical Education, Tehran, Iran

## Abstract

**Background:**

The cesarean section rate has been steadily rising from 35% in 2000 to 40% in 2005 in Iran. The objective of this study was to identify barriers of reduce the cesarean section rate in Iran, as perceived by obstetricians and midwives as the main behavioral change target groups.

**Methods:**

A qualitative study with purposive sampling was designed in which data were collected through in-depth interviews and document analyses. Hospitals were selected on the bases of being public and or private and their response to the ministry's C-section reduction interventions. The hospital director, obstetricians and midwives from each hospital were included in the study. The classification of barriers suggested by Grol and Wensing was used for the thematic analysis.

**Results:**

After 26 in-depth interviews and document analyses, the barriers were identified as: financial, insurance and judicial problems at the *economic and political context *level; the type and ownership of hospitals, absence of an on call physician, absence of clear job-descriptions for obstetricians and midwives, too many interventions in the delivery process and shortage of human resources and facilities at the *organizational context *level; distrust and insufficient collaborations between obstetricians and midwives from macro to micro level at the *social context *level; attitudes toward complications of C-section, reduced capabilities of obstetricians, midwives and residents at the *individual professional *level; and finally, at the *innovation *level, vaginal delivery is time consuming, imposes high stress levels and is unpredictable.

**Conclusion:**

Changing service providers' behavior is not possible through presentation of scientific evidence alone. A multi-level and multidisciplinary approach using behavior change theories is unavoidable. In future studies, the effect of the barriers should be determined to help policy makers recognize the most effective interventional package.

## Background

In recent years the cesarean section (C/S) rate has increased in different parts of the world, both in developed and in developing countries. The rate is considered as an index of healthcare coverage in different nations. While mothers' higher access to healthcare services is linked with the reported increase in the C-section rate, the high prevalence of the condition also reveals that the procedure is performed without any medical indication in many cases. The latter may contribute to higher rates of complications in both mother and neonate [1-3], imposing heavy burdens on the healthcare system [4-7].

In 1985, the World Health Organization (WHO) considered a rate as low as 15% as an acceptable rate for performing C-section [8]. Recent figures released by this organization in 2009, however, revealed that the rate for such a procedure is much higher in different countries worldwide. The Demographic and Health Survey (DHS) conducted in Iran in 2000 reported the C-section rate in the country to be as high as 35% [9]. The 'Integrated Monitoring Evaluation System' Survey (IMES) conducted on urban and rural populations of women aged between 10 and 49 years, as representative of the Iranian population, also reported the rate of cesarean-section to be as high as 40%, which is higher than the rate reported in developed countries such as the US (30.2%) and England (22%). The rate is believed to be comparable with that of fast economic growing countries such as Brazil (41.3%) and China (40.5%). The C-section rate in the neighboring developing countries varies from 4.7% in Azerbaijan to 18.6% in Jordan to 27.6% in Egypt [10]. As a result, the Iranian hospitals were urged to lower the C-section rate in public hospitals to figures lower than 20% and in referral hospitals to less than 25% [11].

The healthcare providers consisting of the obstetricians and midwives along with the pregnant women and their families are the two main target population regarding C-section issues. In each group various strategies should be established to overcome the obstacles encountered in reducing the C-section rate. The most effective intervention, however, should be justified based on efficient analysis of the target population and situations where the change would take place. Concerning this topic, various qualitative and quantitative methods including surveys, interviews and close observations have been introduced [12].

In order to promote safe delivery in mothers, the Ministry of Health and Medical Education (MOHME) in Iran has followed certain interventions in "mother friendly hospitals" such as child birth preparation classes and making rules and regulations for labor wards' standards, but creating behavior change in health care providers is more complex. MOHME has suggested changes in the educational curriculum of midwives and the obstetric residency program, post graduate training courses for obstetricians and midwives and developed guidelines for outpatient and inpatient obstetrical emergencies and has tried to make changes in medico legal and financial issues, but the rate of cesarean section has not reached its goals.

Considering the fact that the barriers of lowering the C-section rate had never been studied in our country, the present qualitative study was conducted to evaluate the barriers of reducing C-section rate in Iran, as perceived by obstetricians and midwives.

## Methods

Two methods were used to collect data in the present study. In-depth interviews were conducted with healthcare providers and the contents of seminars, conferences and health ministry committees held regarding lowering C-section rate were also analyzed. Purposive sampling using maximum variation method was adopted to gather a broad range of information from the healthcare providers. In this regard, hospitals were classified on the basis of being public and private medical centers and based on their response rate to the interventions justified by the government for promoting vaginal delivery. The director of the hospital along with two obstetricians (including the head of the obstetrics department) and two midwives (including one in charge of child birth preparation program) were then interviewed. The reasons for high C-section rate in Iran were the main gist of the questions discussed in the interviews (Additional file 1). Ad-hoc analysis was used to gather more complete data from the in-depth interviews [13]. An oral informed consent was obtained from the participants before the initiation of the interview and they were assured that they could discontinue the voice recording at any point. All interviews were audio recorded and transcribed verbatim.

The researchers had attended several seminars and conferences, the main subject of which was on how to reduce the C-section rate in Iran. These meetings included: A) national committees promoting physiologic labor (including obstetricians and health deputies from different universities to discuss the barriers and solutions for promoting physiologic labor), B) family health seminars, the annual conference of obstetricians and gynecologists (one of the panels of which was devoted to lowering C-section rate in the country), C) national workshops assessing the 5-year program of reducing C-section rate; a two-day program in which a group of obstetricians and a representative of the health ministry, several medical universities, insurance companies, the 'Ministry of Social Welfare and Rehabilitation Services' along with a delegate from the policymaking council of mass media, medical council, and forensic medicine participated to improve their knowledge regarding C-section and ways to reduce its rates. The audio files of A and C were transcribed and the video file of B was evaluated and analyzed by a researcher. The barriers of reducing the C-section rate in Iran were extracted from the content of those meetings.

A thematic analysis was performed [14]. In order to determine the main themes the Grol and Wensing classification, in which the behavioral changes in the healthcare providers were categorized in five main categories of 'economic and political, organizational, social, individual professional and innovation contexts' was used.

In order to improve the reliability of the study, 38% of the interviews (i.e.10) were analyzed by two members of the research team independently. The differences in their reports were assessed and the main themes were extracted

## Results

In total, 26 in-depth interviews were held with the healthcare providers. The number of extracted categories, themes and subthemes are outlined in Table 1. The studied population's quotes are shown in italic.

**Table 1:** Barriers of reducing C-Section rate in Iran; Categories, Themes and subthemes

Categories Theme/subthemes	
Economic and Political Context	
• Economic Issues	Lower tariff for vaginal delivery compared to C-section
	The low share of midwife from vaginal delivery tariff
	Fee for service payment methods
	The role and responsibilities of insurance companies

• Legal issues	Multiple centers for dealing with medical errors
	No respect for the physicians in the court
	Incapability to judge based on the existing condition and available facilities
	Lack of criteria for selecting technicians in the legal system, low knowledge and skill among the technicians,
	The absence of practical guidelines for judges
	Ignoring the stem of the problem
	Inability to produce evidence-based and scientific proofs due to technological problems
	Simplicity of filing a complaint by families
	The absence of any support for midwives
	The high price of blood money

Individual Professional	Physicians' good perception about cesarean section complications
	Physician's declining skills of managing vaginal delivery
	The Teaching residents system
	Midwives' declining skills of managing vaginal delivery
	The teaching midwives system
	Lack of ethics in suggesting cesarean section without indication by providers

Organizational context	Hospital Management Methods (Autonomous Hospitals)
	Hospital type (public, private, teaching or referral)
	Lack of on call physicians
	Lack of physicians and midwives' job descriptions
	Lack of human resources specialized in obstetrics
	Lack of human resources specialized in midwifery
	Additional Interventions
	Unsuitable attitude toward mothers in the labor Room lack of acceptable facilities of the labor Room
	Lack of suitable pregnancy care provision

Social context	Lack of cooperation at associative levels between midwives and specialists
	Distrust between midwives and specialists in hospitals

Innovation (vaginal delivery)	Time consuming
	High stress levels
	Being unpredictable

### Economic and Political Context

This category consists of policies, laws and payments in the healthcare system that affect the behavior of healthcare providers.

### Economic issues

Economic issues were considered as one of the most important barriers faced by specialists. Many of the specialists believed the lower tariff set for specialists in charge of vaginal delivery increased their tendency towards performing C-sections. Some of them also claimed that the fee paid for vaginal delivery is not worth the time consumed and stress endured during such a procedure.

Changing the tariff imposed on vaginal delivery may be one of the strategies adopted by the policymakers to lower C-section rate. Specialists however, have controversial opinions regarding the effect of such a change on the C-section rate. Some specialists who participated in the study said the tariff imposed on vaginal delivery in Iran should be equal to that of C-section in order to adjust the C-section rate. Others however, believed the tariff imposed on vaginal delivery should be two, three or even five times higher than that of C-section.

The majority of specialists considered the low tariff imposed on vaginal delivery compared to C-section as the main obstacle in achieving such a goal. Many of them however, did not believe increasing the tariff would solve the problem.

"I don't care about the tariff; I really don't have time for it (vaginal delivery). This is of great concern for me as the procedure (vaginal delivery) is really time-consuming."

"I won't do it (vaginal delivery), even if I'm paid 10 times more."

Another physician said considering the covert relation between the patient and her physician, changing the tariff would be of no good in reducing C-section rate.

On the other hand, setting tariffs for labor affects the relation between physicians and midwives. One of the physicians added that,

"Many midwives claim that physicians receive all the money so why should a midwife spend long hours in the labor room; physicians, on the other hand, claim they should receive more money as they are in charge of any possible legal problems linked to labor."

Various issues have been addressed regarding the effect of money concerns on the motives behind the midwives getting involved in vaginal delivery. Many of the midwives are not paid per case; however, there is no agreement regarding the effect of not paying midwives for the labor on their intentions and function. A midwife who really loved her profession claimed that

"we should not stop working due to the existing problem and the fact that we are not paid for our job. I should do my job accurately but urge policymakers to pay me correctly. However, as the physicians are responsible for the whole event and they should respond in case a legal problem occurs they should receive more. Midwives could manage hard labors previously but now they can't as they avoid such situations due to financial problems."

Another midwife said,

"Modifying the paying system is a crucial solution for this problem; the whole team, not a single person, should be paid for the job."

One of the specialists claimed that changing the payment method is an important strategy in achieving such a goal:

"The paying system should be changed completely. Paying physicians a definite salary rather than based on the number of cases they visit, would change the condition significantly. If the physician is forced to stay in the hospital for certain hours, she becomes responsible for patients she visits and thereafter she is paid for that, rather than the fewer or more patients she has visited, and then the condition would change."

### Insurances

The payment system is one of the barriers mentioned by the majority of the participants. One of the midwives blamed the payment system, mainly the insurance companies, saying that,

"In Iran, the insurance companies sign a contract with healthcare providers and pay them rather than compensating the service itself. Considering the fact that the service provided by the midwives is not covered by insurance companies, expectant moms prefer to go to a specialist. In this situation the rate of additional interventions and C-sections would increase."

Some of the participants claimed that the insurance companies have lots of power and can therefore influence policymakers of the health system. In 2004, MOHME posted a circular (no S/2/55019) defining a number of indications for C-section. Thereafter, the insurance companies limited the payment of C-sections to the mentioned indications. Some of the participants, however, claimed that the circular had been prepared under the influence of the insurance companies.

The limitations imposed on paying for C-section by insurance companies have not reduced the number of such procedures. The economic relation between the patient and her physician, which forces the physician to get paid by the patient rather than the insurance company, was one of the main reasons contributing to this fact.

Many physicians believe the limited number of indications for C-section accepted by the insurance companies has contributed to major problems. Not recording the exact indication leading to C-section in the patient's medical record is one of the main concerns in this regard. In other words, some physicians document a reason accepted by the insurance company in the patient's chart, making the accurate assessment of the underlying reason for C-section rather impossible.

One of the problems caused by the release of the circular is an increase reported in the mortality rate among pregnant women. One of the specialists claimed that,

"Reducing the number of C-section has turned into a threat for pregnant women. The MOHME circular has caused a similar effect. Non-responsible personnel such as the head of the network and health officials in small provinces force young specialists to stay away from C-section, subsequently causing a considerable increase in the mortality rate among pregnant women."

One of the specialists claimed that the fact that supplementary methods are not included in the insurance tariffs accounts for the increased rate of C-section in a special group of mothers in the country. She added that families pay large amounts of money for the pricey service and therefore look for more safety measures.

Medical malpractice insurance is another factor, which many specialists believe, acts negatively for physicians and midwives. One of the specialists stressed that

"The absence of medical malpractice insurance has caused various problems for midwives, damping their enthusiasm for having an active role in labor."

Another participant added that

"Biased physicians can continue their erroneous work thanks to the medical malpractice insurance."

### Legal issues

Some of the proclamations made by the physicians revealed the importance of legal issues in increasing the C-section rate:

"Being brought to court, even once, makes the physician and her near friends keep away from vaginal deliveries for ever. In the court, they behave rudely towards the physician, making her behave in a similar manner towards others."

"The obstetricians are always blamed unless the opposite is proved."

One of the problems faced in this regard is the disrespectful behavior with the physician in these situations. One of the participants claimed that

"The court treats physicians like other culprits."

One of the specialists stressed that a patient can file a complaint with three officials including medical council, forensic medicine and a special court in judiciary, making the physician's condition worse. The family and the child can also lodge a complaint against the deliverer even years after the labor, a situation which intensifies the physicians concerns in this regard.

Another problem pointed out by a specialist was the incapability of judges in such conditions:

"We can't compare the health system in Tehran (as the capital of Iran) and that of Zabol (a city with limited resources), but when someone dies nobody checks to see whether the death has occurred in Zabol or Tehran. They believe Zabol should have the same resources as Tehran."

Some of the participants consider the high price of blood money as another setback for the judiciary system. In other words, considering the fact that a physician is always responsible for the complications faced during a vaginal delivery and the high blood money rate, many physicians prefer to do a C-section which is safer.

Judges decide based on their technical consultant's point of view, many of whom lack sufficient knowledge and skill for detecting medical errors. Some specialists claimed that the technical consultant, selected mainly based on relations, do not seek the underlying cause responsible for the complication and let their emotions decide.

The head of a hospital, who was a law technician himself, stressed that he did not agree with his friends, saying that,

"Judges are skilled and study their dossiers with great attention."

There is, however, no guideline or scientific basis which would guarantee the judging process. The specialists believe the vaginal delivery may be accompanied with certain complications based on scientific evidences, and these have nothing to do with the specialists' skill. the fact that the judges do not hunt for the underlying cause of the complication reported during a vaginal delivery is among the problems mentioned in this section. One of the midwives said:

"The judiciary system receives a dossier on the death of a patient due to massive bleeding during vaginal delivery. The judge rules that the patient should not have undergone NVD, without asking why she had received induction while only a short time had passed since her EDC (Estimated Date of Confinement)."

On the other hand, lack of accurate technology (fetal monitoring systems), capable of providing acceptable evidence for judges, are among other problems faced in this regard. One of the specialists stated that,

"Having an ABG device for assessing the fetal umbilical cord blood in each hospital can provide the physicians with definite evidences about the presence of asphyxia in the newborn."

Mothers asking for a C-section were among other issues mentioned by the specialists and midwives. The interaction between the family demand and the weakness of the judiciary system was highlighted in these sayings.

*"Many mothers insist on undergoing a *C*-section from the beginning of their pregnancy. If a vaginal delivery would become problematic in such patients, they would file a complaint against the physicians. Many physicians, therefore, do what their patients ask of them as courts support patients rather than physicians in the majority of the cases."*

Some of the participants considered public unawareness and the easy and cheap process of filing a complaint as the factors accounting for the increased number of complaints against obstetricians.

Some specialists claimed that many families receive a certain amount of money from the physician to withdraw the legal process, stressing that it not only reduces the physicians' tendency towards performing a vaginal delivery but also tempts other families to file similar complaints. One of the midwives noted that

"Our society has spent more time on teaching the process of suing rather than introducing the labor to the general public".

Other midwives stressed that the fact that the midwives are not actively involved in the vaginal delivery has contributed to such legal problems. Midwives believe they are not supported by law. One of the physicians noted that during a vaginal delivery midwives come to a decision that performing a C-section is inevitable often too early and without sufficient evidence and as this claim is recorded in the patient's medical record, the physician is afraid to give the mother more time to deliver her baby physiologically. In this regard, a midwife, however, responded that,

"Midwives decide in favor of C-section whenever a minor problem occurs as they are dealing with feelings of job insecurity. They are afraid to be taken to a court for problems caused during a vaginal delivery. The law does not protect midwives. Physicians are more protected by law."

Having a national 'midwifery and delivery clinical guideline' in "Mother Friendly Hospitals" as a legal reference, standardizing the criteria for selecting the court technicians (an academic staff with at least 10 years of experience in the field of delivery and C-section who has passed the relevant educational courses), developing a legal guideline on delivery problems, the presence of Ob-Gyn residents in the legal court sessions, establishing a legal consulting company for guiding physicians and midwives in the medical council, and rewriting Oncall laws were among the solutions mentioned in this respect. Considering the fact that confining the time in which families can file a complaint is rather impossible, an unconstrained medical malpractice insurance package especially meant for delivery problems should be prepared for the physicians.

### Individual professional

This section aims to evaluate the knowledge, perspective, awareness and motivation of healthcare providers for their behavioral changes and factors affecting them.

The perspective of the specialists regarding the complications of the vaginal delivery compared with that of C-section plays an important role in determining their tendency towards performing each of the operations. Some specialists believed C-section is associated with serious complications, whereas others claimed the complications secondary to C-section are ignorable. Some of the participants said that the C-section complications are not more severe than that of the vaginal delivery, adding that C-section reduces the risk of developing CP in the newborn. One of the specialists stressed that,

*"*C*-section for multiparous women is associated with limitations and various complications but if the mother intends to have a single or at the most two deliveries not many complications arise."*

Another specialist, however, said that

"Despite the reduced number of pregnancies, women undergo surgeries due to various other reasons in which the adhesions caused by previous C-sections might become troublesome."

On the other hand, many physicians believe the process of vaginal delivery is often difficult and unacceptable. They believe

"Vaginal delivery is performed in a bad condition in our country is."

Many do not believe in the C-section-associated complications; one of the specialists, therefore, claimed that

"insurance companies are setting up all the propagandas against C-section to pay less. These companies advertise for vaginal delivery in order to pay less for delivery."

The specialists' point of view regarding "ethical issues" was another factor mentioned by two of the participants.

"The physicians should respect ethical rules. They should avoid C-section whenever there is no indication for such a procedure. For instance, if a woman wants to ligate her tubes, they recommend her to undergo a C-section to have a TL,"

one of them said. One of the midwives similarly pointed out the impact of ethics and dedication on the healthcare providers' behalf, saying

"Paying the tariff to the specialists who have been only in charge of the delivery and have inspired the mother to undergo a C-section is the result of not respecting the ethical laws."

The majority of the participants believed that the capacity of the Ob-Gyn specialists and residents in conducting a NVD has reduced in recent years, adding that the increased number of universities and teaching hospitals as well as the increased number of Ob-Gyn residents has led to the poor quality of education and subsequently economic problems for physicians.

"Previously the complicated patients were transferred to referral hospitals and visited by special physicians. Nowadays all the hospitals have become a teaching hospital and Ob-Gyn residents therefore rarely visit complicated cases."

"In the past few years many obstetricians have never had the opportunity to do a vaginal delivery. The knowledge of a first year resident regarding the procedure is similar to that of an intern. Residents learn the process of natural delivery during the first year but by the time they have learned how to deal with physiologic labor, the year ends and a new unskilled group becomes responsible for the whole thing."

Although there are exact numbers in the residency log book that should be done for every procedure including NVD, those are neglected by the professors because of lack of time and cases

"A residency course may last more than 20 years if we ask a resident to perform a specific number of procedures before graduating."

Similarly, a midwife claimed that

"Many first year residents transfer mothers from labor rooms for a C-section as they need to learn C-section before entering the second year."

Midwives, who are responsible for controlling and performing a natural delivery, play a critical role in promoting physiologic labor. Many physicians believe having a motivated midwife involved in the system is of great importance. During the past years, however, midwives have faced many problems as many of them play the role of a nurse or a secretary in the hospital. This along with other factors lowering the motivation and capability of the midwives and midwifery students in the related fields has accounted for their less active involvement in natural delivery and subsequently a decline in the quality of their education.

Some of the physicians claimed that increasing the capacity of the universities along with the erroneous and unwritten laws in the educational centers play a critical role in reducing the quality of midwifery education. The director of one of the hospitals stated that,

"In our hospital, obstetricians are responsible for delivering the baby as it is a teaching hospital and Ob-Gyn residents should learn such procedures. The greatest challenge for me is the fact that my obstetrician colleagues try to dominate the whole delivery process, undermining the role of the midwives, who should be responsible for the whole process. If you ask any of the midwives in our hospital, they attest that they have not conducted a natural delivery for years."

Similarly, one of the midwives added that,

"Nowadays, filling the quota is the only important matter. A large number of students are graduated in the field of midwifery from different universities across the country, regardless of their facilities quota. Many of them only observe the delivery process to complete their quotas, as the university has told them to find a field for themselves."

### Organizational context

In this category, variables such as the care process, human resources, capacities, economic resources, and the structure of the healthcare providers and organizations are discussed.

### The Hospital Management process

One of the midwives said,

"The autonomy plan has deeply damaged the health care system as these independent hospitals do anything to have higher incomes. The lower tariff imposed on vaginal delivery compared to C-section and the fact that the midwives receive less than physicians accounts for the visits performed by a midwife; there is now a tendency towards providing more specialized service."

It should be stressed that a certain number of hospitals in Iran are autonomous organizations, responsible for their own expenses.

### Oncall Physicians

Several participants considered the absence of an on call physician as an obstacle in the way of performing natural delivery, and subsequently the underlying cause of increased C-section rate.

One of the specialists stressed that

"The absences of on call physicians in big cities such as Tehran, where there are long distances between houses and hospitals and there is always fear of traffic and being late, have made physicians perform more C-sections. The presence of an on call physician in the labor department, therefore, is needed."

In this regard, many of the physicians claimed that the presence of an on call physician in the labor department should be considered as an index in hospital evaluations. In the absence of on call physicians, on the other hand, residents are responsible for managing the labor. One of the specialists claimed,

"The absence of specialists in teaching hospitals is another problem. Residents who perform the job, decide in favor of C-section as soon as even a small problem is encountered due to their lack of experience. They call the professor, who is at home, and she takes up the resident's decision of performing a C-section. A skilled physician should always be available in the hospital."

### Types of hospitals

Some specialists believe the type of hospital -- public or private, teaching or referral -- plays an important role in determining the C-section rate in different ways. They added that a public hospital, known as a referral center and located in the center of the city, admits patients from different parts of the city and as the majority of the referred cases are believed to be difficult and complicated, a higher rate of C-section is expected to be recorded in this center. As a result, the rates should not be compared between hospitals of different types. It should be stressed that the patients admitted at referral hospitals have already received certain care in a medical center, although unsuccessful, and therefore they should undergo a C-section without wasting any time.

One of the midwives claimed that the high load of work in the labor rooms is another factor contributing to the increased c-section rate. She added that

"our center is too crowded and this is an important factor. We send expectant mothers who can be C-sectioned rapidly to the operation room in order to have more vacant beds."

In teaching hospitals, yet, other factors may influence the whole process

"The absence of full-time specialists in teaching hospitals and the fact that 1st and 2nd year residents are responsible for the delivery and that the midwives are marginalized in the whole process have contributed to an increase in the C-section rate in these hospitals. The situation may increase the total C-section rate in the country through influencing the educational system in the residents and the midwives."

### Additional interventions

The majority of the specialists claimed that medicinalizing the process of labor and adding interventions (such as hospitalizing, maintaining an IV-line and injecting solutions, elective induction and frequent vaginal examination) are among the factors turning physiologic labor into a non-physiologic process and consequently increasing the C-section rate. The absence of a scientific and accurate hospital protocol has also contributed to the addition of unnecessary and often non-scientific interventions to the labor process.

Some of the midwives considered the early admission of mothers at the time of delivery as the reason of additional and unnecessary interventions, and consequently C-section in certain cases.

"An expectant mother who is being monitored is confined to bed and this makes her intolerant. She is receiving IV-solutions and so is not permitted to go to the restroom as she is catheterized. These unnecessary interventions increase the risk of C-section."

Some of the midwives added that induction in patients with no evidence-based indication may also increase the C-section rate,

"Induction is equal to increased C-section rate."

One should pay more attention to the underlying reasons of these additional interventions. One of the midwives said

"A single midwife was responsible for the night shifts of a village. It is understandable that she had to carry out certain interventions including induction to lower the burden."

### Job description for the specialists and midwives

Another problem mentioned by the majority of the participants was the absence of a precise job description for the specialists and midwives during the labor process. In the absence of such a guideline, it's not clear when the physician should take the responsibility for the process and who is to blame if and when a problem occurs. Considering the reported legal issues, the specialists and midwives both prefer C-section.

Some specialists stressed that developing a job description for the midwives has various benefits, as it boosts teamwork in the labor process. They however added that while sharing the responsibilities is important for achieving the final goal, it should be done based on scientific evidences and concerns about the economic issues. In this regard, some specialists believed that natural delivery should be performed by a midwife under the supervision of a specialist.

One of the midwives stressed that involving the midwives in the labor process and encouraging teamwork can help reduce the C-section rate,

"In private sections we can have a team consisting of several midwives and an obstetrician. An on call midwife can accompany the expectant mother in pain to the hospital, so there would be no need for me to tell the mother when it's time for her to give birth. The hospital staff should cooperate with the midwives and follow their orders. The midwife can have the main role in the labor process unless the patient asks to have a physician at her bedside. The midwife should then be paid based on the tariff set for an on call midwife. In these situations the physician only interferes if a problem occurs. The specialist can also ask a midwife to stay at the bedside of her own patient until it's delivery time and thereafter the physician can carry out the process herself."

### Lack of specialized human resources

Lack of specialized human resources has also led to the increased C-section rate in some cities. The only obstetricians in such locations prefer to lower the risk of emergency by performing pre-programmed C-section in the limited number of days they are in the city to avoid stressful on call nights.

### Lack of midwives

Lack of midwives may contribute to increased C-section rate in different ways. It makes not only the delivery process dependent on medical instruments but also the expectant mother to feel alone in the labor room, making the process more intolerable. Some mentioned that

"We need more resources. I can't ask my resident (in teaching hospitals) to stay at a single patient's bedside for the whole process as she is responsible for the numerous complicated deliveries which occur in our center."

"In places where the C-section rate is low, there is a single-bed labor room and a particular midwife who stays with the patient until the end of the delivery process."

### How to deal with a pregnant woman during a natural delivery

Some specialists believe the way personnel treat an expectant mother during labor influences her decision about reselecting that type of delivery in the future or recommending it to her friends.

"In Iran, a midwife or resident is responsible for a couple of patients. Several patients are examined during each shift. As a result, many of the patients are against undergoing natural delivery, particularly in a non-private hospital. Many mothers, with a previous experience of normal delivery in the private sector ask to undergo a C-section the second time. In other words, our system is not appropriate for normal delivery."

The presence of a companion during the labor and treating mothers nicely can also help tackle the obstacles in this regard. One of the midwives stressed that,

"The companion talks with the patient and this reduces the patient's stress. They go to the next step together gradually. But considering the fact that we don't have enough human resources in the field, the quality of communication between the midwife and the mother has declined."

The companion in the labor room can be one of the relatives or a midwife who has accompanied the mother during the pregnancy. She, however, should be well-educated so that would not meddle in the process or increase stress. In this regard, specific regulations on the topic of the legal responsibilities of the patient, midwife and the hospital have been established.

One of the physicians noted that,

"I decided to take the midwife who worked in my clinic to help me through labor in a private section. I promised to pay all her salary myself. My patients had formed strong emotional bonds with my midwife during the 9-months they had visited my clinic for prenatal care. I didn't want a stranger midwife help me with the process. The hospital, however, did not accept, saying this would cause a chaos in the system. Whenever you try to modify the system you face a problem."

### The facilities and physical environment needed for natural delivery

Some physicians claimed that the physical environment of current labor rooms is far from standard. It is clear that such unsuitable conditions would negatively influence the mother's perspective and subsequently her decision regarding her type of delivery. These physicians stressed that,

"The labor rooms are not in a good condition; they should be equipped with clean restrooms and baths, so that expectant mothers are able to take a bath whenever they need to. There are no pillows in our department. It is not possible to promote physiologic delivery without spending on it. Large sums of money are needed to equip and decorate the labor rooms and to add rest rooms to them. In our department, rest rooms are placed at the other side of the department; the patient is forced to use the basin in front of others, a disgracing condition."

One of the midwives claimed that the standards of the labor rooms have changed over time in order to reduce the rate of natural delivery. She added that,

"Contrary to international standards, the size of our labor rooms have reduced and they have been converted into operating rooms over time."

One of the physicians stressed that in order to solve the problem

"The facilities and human resources in these rooms should be standardized. These facilities are old fashioned and designed for group labor rooms, and therefore should be modified."

### Absence of labor care

While the national healthcare system is, to some extent, responsible for the absence of prenatal care in many places, lack of knowledge in many families and their inability to visit healthcare providers are among other factors contributing to the tragedy. One of the specialists added that,

"Patients do not receive the required care during pregnancy and therefore the high-risk cases are not detected. Some of the patients admitted at the hospital with pre-eclampsia and edema have not been assessed during their 9 months of pregnancy, and should undergo a C-section according to the current laws and text books."

### Social context

This section discusses the influence of the professional point of view and team working culture and the leadership behavior on the performance of the healthcare providers. Obstetricians and midwives are considered as the two main arms of the delivery process but unfortunately they do not cooperate ideally with each other. One of the midwives stressed that

"There is no joint meeting between the midwifery and obstetricians associations."

One of the specialists added that

"Trust issues between the midwives and specialists are a source of defect in the system."

It should be noted that one of the specialists, who did not agree with this point of view, stressed that trust issues are no more than an excuse for physicians to refuse involving midwives in the process.

One of the midwives added that the midwives and specialists also fail to collaborate due to the discrepancies found between their scientific evidences,

"The discrepancy between the midwives' and the specialists' information is our main problem. We don't believe in issues that the physicians accept as true. We do our best to make physicians accept our proposals in certain cases but the residents change frequently before winning our trust. In other words, too much time is needed before the physicians would accept our proposals and therefore we have to work gradually. We, however, do our best."

### Innovation

This category aims to discuss obstacles such as feasibility, acceptability, and creditability in promoting a new behavior, for instance increasing natural delivery and reducing C-section rate in the target population.

Compared with C-section, natural delivery is associated with more complications. According to the participants, the process is more time consuming, more stressful, unpredictable and disturbs night sleeps.

"The main problem with natural delivery is its unpredictability, as it may occur anytime and disturb the physician's program. For example, the physician goes to the hospital in the morning and to the clinic in the afternoon. But she has to return to the hospital in the afternoon to carry out a natural delivery. In the first ten years of my own experience, I accepted to perform such deliveries. But now I work the whole day and reach home at 10 pm. I can't revisit my patient in the hospital at 10 pm to carry out a vaginal delivery."

### Maternal features

This category accounts for patient-related variables such as knowledge, skill, attitude and capacity.

Some specialists claimed that one of the factors affecting the rise in C-section in the country is that the mothers and their families ask for a C-section.

"Mother's unawareness is as harmful as the physician not willing to do a vaginal delivery,"

said a participant, pointing out the importance of such demands in the physicians' point of view.

### Awareness and perspective

One of the participants stressed that maternal unawareness regarding labor along with mothers' imprecise knowledge about different delivery methods, their complications, and their hospitalization period has reduced their tendency toward undergoing a vaginal delivery. She added that,

"A mother who enters the system is concerned about the delivery method, the status of her fetus and whether he/she would be healthy after delivery. She is worried about the influence of labor on the unborn and herself."

Inaccurate advertisements ran by the media, bad experience described by other people or even the mother herself, the governing belief in the society and not providing the patient with the accurate information by the physician are among factors leading to low public awareness in this regard.

"The mother's perspective in this regard is of great importance. The general belief indicates that C-section is better than vaginal delivery. The dominant paradigm says so. The most important individuals who prompt such a belief are the specialists."

One of the midwives stressed that

"Evidence-based medicine, which we are trying to follow in our practice, stresses that one of the vaginal delivery complications is the relaxation, but do we inform our patients about the complications associated with C-section as well? Never. Do we inform mothers about possible side effects of the anesthetic agents, injuries sustained to the genitourinary system, more bleeding, higher infection rates and more infant-related problems associated with C-section? The husband calls for a C-section as soon as we tell them she would be relaxed in this technique. In a study conducted in Iran, 87% of the individuals who sign the consent for the C-section surgery were not aware of the content of the form."

Another midwife added that,

"Media significantly influence the public belief about medical issues. An obstetrician is invited to a program to discuss simple topics such as labor. Pediatrics, similarly, are asked to talk about common cold in an infant."

One of the specialists stressed that a woman and her husband's awareness about the genital complications of natural labor (pelvic relaxation) and its effect on sexual relation along with the fact that law does not protect women with such disabilities (religious laws allow men to remarry and have multiple wives) has made families have a higher tendency toward C-section.

"Mothers ask for a guarantee for not developing prolapse if they undergo a vaginal delivery."

### Maternal capabilities

One of the physicians claimed that

"The modern lifestyle and the anatomical differences between Iranians and those from other countries have affected the former group's capability to undergo vaginal delivery. The sedentary lifestyle and not following a healthy diet have reduced the capabilities of our girls in this regard."

One of the midwives working in the private sector said mothers play an important role in choosing the delivery method. Lifestyle has affected maternal capability, making a vaginal delivery more difficult for them. She considered

"Not receiving the related educations or getting involved in certain exercises as the factors contributing to this finding."

### Maternal Capacity

The majority of the physicians and midwives believed fears of labor pain have increased the mothers' tendency toward C-section. They said one of the ways to tackle the fear is to teach mothers about the real nature of these pains.

On the other hand, reduced number of pregnancies as well as the increased age of marriage and pregnancy is among other factors leading to the families' higher tendencies towards undergoing a C-section.

One of these physicians stressed that

"lack of trust in the physicians accuracy and on time decision making is another factor forcing mothers to undergo a C-section. As a result, we should assure mothers that C-section would be performed if needed, adding that vaginal delivery would not be our choice if its risks outweigh its benefits. In other words, we choose the method which is best for both the mother and baby."

### Cultural Issues

Cultural differences in various societies also determine the mothers' tendency toward undergoing a vaginal delivery; these effects are more pronounced in certain cities. In some parts of the country, husbands do not permit their wives to undergo a C-section even in critical conditions; in some cities, however, women are considered as working forces both inside and outside the house and families, hence, vote in favor of vaginal labors so that the mothers would return to their daily routine in the shortest time possible. In these nations, C-section is a failure for women. In certain other nations, however, C-section is the preferred technique,

"C-section is becoming more common and stylish these days."

One of the midwives added that, "Cultural issues are the main obstacle for many Iranians. Being capable of doing more vaginal deliveries is embarrassing and the educated class therefore, mainly undergo C-section."

The participants stated various solutions, including educating mothers and their families, changing their point of view about midwives, promoting vaginal delivery by holding educational courses for mothers, highlighting the benefits of vaginal delivery, reducing fears of natural labor and promoting pain free delivery, drawing the public attention to the complications associated with C-section, permitting the presence of a companion during vaginal delivery, increasing awareness by mass media, improving general public's awareness and knowledge on the topic, and educating male teenagers about their role as a father in some 10 years in schools.

## Discussion

The present study discussed the perspectives of obstetricians and midwives regarding the barriers and solutions of reducing the C-section rate in Iran. The detected obstacles were then classified into different categories according to the Grol and Wensing classification. As mentioned earlier, various barriers interact with each other at different levels, creating a complex atmosphere in which the recognition of factors effective in reducing C-section rate becomes more difficult. Among healthcare providers, the barriers were found at personal level (awareness and skill of specialists and midwives) to organizational level (the absence of on call physicians, performing too many additional interventions, factors related to mutual cooperation between specialists and midwives, labor room standards) to policymaking level (the payment method, legal issues, educational system and lack of human resources); ignoring any of these factors would make the reduction of C-section rate impossible. Moreover, healthcare providers claimed that mothers' tendency towards undergoing a C-section was one of the main problems against reducing the number of C-sections in the country. They added that the absence of scientific-based guidelines for judgment and the mother's tendency towards C-section are the main reasons troubling physicians by legal issues in this field. Specialists particularly pointed out their lack of trust in midwives abilities in managing natural delivery. Midwives believed the c-section rate had increased after the responsibility of delivery had been taken away from them; they also pointed out the unsuitable pay they receive per delivery.

Aiming to change the behavior of the healthcare providers, particularly specialists, various interventions have been performed in different parts of the world; including the development of audit and feedback, mandatory second opinion, quality improvement (one to one nursing care, standardization of the criteria used to determine the initiation of labor, labor management based on clinical guidelines, electronic monitoring of fetal heart, using intra-uterine catheters to determine uterine pressure, promoting vaginal delivery after a C-section, developing midwifery teams) along with multifaceted interventions (using two or more interventions targeting different populations), and provision of prenatal and labor care by a midwifery team [15-22]. A review article showed that multifaceted interventions were more effective in reducing C-section rate if audit and feedback were performed [23]. It should be mentioned that the majority of these interventions are set at the organizational level and target the care providing system. The present study, however, revealed various barriers, each in need of specific solutions, faced in the way of reducing C-section rate at personal and policy making levels in our country. Considering these barriers, certain solutions should be obtained to achieve the final goal.

As for obstetricians, educating them in order to change their attitude towards C-section complications and improving their skills in performing vaginal delivery should be addressed. Changing their perspective on the indications of C-section is also necessary [24], as choosing the best delivery technique depends on the physician's judgment and as mentioned earlier some of the criteria (pre-eclampsia and edema) currently used for doing a C-section are not scientifically supported. Changing the educational rules for residents also play an important role in increasing their tendency toward performing vaginal deliveries. Many residents imitate the specialists' behavior so reducing the C-section rate is only possible when the principle obstacles in the specialist are addressed. improving the legal system for increasing specialists' motivation for doing vaginal deliveries is also very important. Complaints against obstetricians account for 8% of the complaints filed with medical council judiciary; physicians were considered guilty by the forensic medicine in 40% of these cases [25]. Using a unified center for dealing with these complaints, changing the judges and organizations viewpoint towards physicians, and establishing practical scientific guidelines for judging are among necessary solutions in this regard. Moreover, providing acceptable labor-related evidences based on the suitable technology for the judiciary system and revising the complaint laws is necessary.

In line with previous studies [26], some participants stressed that making the tariffs imposed on vaginal delivery similar to or higher than that of C-section is not an effective solution in this regard, at least as the first step. Considering the report on modifying the Iranian healthcare system, published by the World Bank, the adaptation of fee for service payment method by insurance companies has led to the implementation of unnecessary service [27] in different procedures such as C-section. Moreover, the fact that user fees and insurances are the main sources of income in self-governing hospitals [28] has increased the tendency towards C-section in these hospitals. As a result, modifying the payment method can help effectively reduce C-section rate. In addition, changing the tariff imposed on these procedures as well as clarifying the amount of money the specialists and the midwives receive for the care they provide is among principle points needing further revision in this regard.

An appropriate distribution of obstetricians in the country is another solution for the problem.

As for the midwives, however, the condition is much more complicated. Compared with specialists, the midwives have a much more positive perspective toward the benefits of vaginal delivery. Their motivation and practical skills in this regard, though, is a matter of concern. In the majority of the hospitals, specialists are responsible for vaginal delivery and the tariff set for the procedure, thus, belongs to them. Not being responsible for the event and not receiving enough money for doing the job are the two main factors affecting the midwives motivation towards getting involved in the vaginal delivery process. It should be noted that making them responsible for vaginal delivery and increasing their payment for performing the procedure are not effective strategies until the quality of education in midwives is improved. Accordingly, improving the educational condition for midwives through improving the quality of education in the universities and reducing their admission capacities at the same time can help reduce the C-section rate effectively.

It should be noted that while the capacity of midwifery schools has recently increased, the hospitals still deal with midwife shortage, a condition which needs further revision of human resources allocation standards and the modification of related employment rules.

Having an on call physician in the hospital can reduce the C-section rate only if the specialist trusts the individual and she receives sufficient salary, or else it is possible that she would assign mothers for C-section with no scientific reasons.

Vaginal delivery is the result of a joint effort in which mothers, specialists and midwives are involved. The knowledge and skill of the team members and their cooperation guarantees the outcome of the job. The specialists and midwives should trust each other and the system to improve the quality of the teamwork, and this cannot be achieved unless better laws are adopted in which the job description of midwives and specialists and the time the responsibility is shifted from the midwife to the physician are clarified. The economic issues should also be addressed in the law, which its feasibility requires the accurate recording of the events and the usage of the evidences by the judicial system.

The admission of mothers before the initiation of active labor may increase the number of unnecessary interventions. As a result, a feasible, practical and scientific protocol accepted by the country's judicial system is needed to prevent the additional interventions from the admission time.

Standardizing human resources in the labor room and improving the physical condition of the place along with the presence of an expert companion during labor can not only make the process more pleasing for mothers but also influence her decision for determining the delivery technique in future pregnancies.

Determining the optimal C-section rate in the country and in referral hospitals is of great importance. There is no agreement over the standard C-section rate across the world [29,30] and the rate should vary in hospitals with dissimilar facilities. As a result, in order to compare and evaluate different hospitals, the rates should be calculated and interpreted for each hospital based on its level of care condition and the population it covers.

One of the main limitations of the present study was the fact that it had only assessed the point of view of a group of stakeholders: healthcare providers. It is obvious that gathering information from the expectant moms and the society would help policy makers learn more about the problem and its solutions.

## Conclusions

Changing service providers' behavior is not possible through presentation of scientific evidence alone. The multi level and multidisciplinary approach using behavior change theories is unavoidable. The present study pointed out that C-section rate reduction cannot be achieved in short term and with blind interventions. Further studies should assess the effect of each recognized barrier in this study before designing short-and long term interventions or the suitable package for reducing C-section rate. The interventions should be chosen with great caution in order to reduce the C-section rate safely and without placing the mother and unborn at risk of death or at-birth complications.

## Abbreviations

DHS: Demographic and Health Survey; IMES: The Integrated Monitoring Evaluation System Survey; C-Section: Cesarean Section; MOHME: Ministry of Health and Medical Education; Ob-Gyn: Obstetrics and gynecology; CP: Cerebral Palsy.

## Competing interests

The authors declare that they have no competing interests.

## Authors' contributions

BY contributed to the design, performed the interviews, analyzed and interpreted the interviews, wrote the draft and discussion, and revised the content. RM, SN, AR and NCH have contributed to the design, interpretation, and discussion. KM has contributed to the design of the study. All authors read and approved the final manuscript.

## Authors' Information

Dr Nasrin Changizi was the head of Maternal Health Office of MOHME at the time of designing the project.

## Pre-publication history

The pre-publication history for this paper can be accessed here:

http://www.biomedcentral.com/1472-6963/11/159/prepub

## Supplementary Material

Additional file 1 **In-depth interview guide**. This guide contains structured questions asked from obstetricians-gynecologists and midwives by the interviewer in order to Identifying barriers of reducing the rate of cesarean section in Iran. Click here for file
