# Supplementary material for: Cesarean section rate in Iran, multidimensional approaches for behavioral change of providers: a qualitative study
Source: BMC Health Serv Res. 2011 Jul 5;11:159. doi: 10.1186/1472-6963-11-159 (PMC3146409; doi:10.1186/1472-6963-11-159)
Supplement: Additional file 1 — In-depth interview guide. This guide contains structured questions asked from obstetricians-gynecologists and midwives by the interviewer in order to Identifying barriers of reducing the rate of cesarean section in Iran. [file 1472-6963-11-159-S1.DOC]

**Additional file 1**

**In-depth interview guide**

**Identifying the barriers of reduce the** rate of cesarean section in Iran

| **Introducing the study to the interviewee**  The objective of this project is to identify the barriers of cesarean section reduction in Iran as perceived by obstetricians and midwives.  This study is being conducted in collaboration with the ministry of health and medical education.  The interview will take about 60 minutes. Since I do not want to forget any of your valuable comments I would like to take permission to audio-record it. If there is any part of the interview that you do not want to be recorded it will not be. So please let me know when to pause the recording. |
| --- |

1. Why do you think the rate of cesarean section has increased in recent years?
2. Which mode of delivery do you think is better, natural or cesarean? Why?
3. Do you think the rate of cesarean section should be decreased in the country?
4. Which do you think have contributed more to the rise in c-section rate, service providers (physicians and midwives) or service receivers (pregnant mothers)?
5. How do you think the increased tariff for cesarean has influenced the rise in c-section rate?
6. In relation to unintentional medical error during delivery, what deficiencies do you think the judicial laws have?
7. What solutions can you propose for c-section reduction? Where should one start from?

Interviewer’s guide: Question 8 should be asked from the Hospital Director or Head of The Gynecology and Obstetrics Department.

1. What do you think the proportion of natural vaginal delivery’s tariff should be compared to cesarean to encourage specialists to perform natural delivery?

Interviewer’s guide: Questions 9 should be asked from midwives alone.

1. How do you think the tariff for natural delivery should change to encourage you to perform natural delivery?
